# In Vivo Optogenetic Control of Striatal and Thalamic Neurons in Non-Human Primates

**DOI:** 10.1371/journal.pone.0050808

**Published:** 2012-11-30

**Authors:** Adriana Galvan, Xing Hu, Yoland Smith, Thomas Wichmann

**Affiliations:** 1 Yerkes National Primate Research Center, Emory University, Atlanta, Georgia, United States of America; 2 Morris K. Udall Center of Excellence for Parkinson’s Disease Research, Emory University, Atlanta, Georgia, United States of America; 3 Department of Neurology, School of Medicine, Emory University, Atlanta, Georgia, United States of America; National Institute on Aging Intramural Research Program, United States of America

## Abstract

Electrical and pharmacological stimulation methods are commonly used to study neuronal brain circuits *in vivo*, but are problematic, because electrical stimulation has limited specificity, while pharmacological activation has low temporal resolution. A recently developed alternative to these methods is the use of optogenetic techniques, based on the expression of light sensitive channel proteins in neurons. While optogenetics have been applied in *in vitro* preparations and in *in vivo* studies in rodents, their use to study brain function in nonhuman primates has been limited to the cerebral cortex. Here, we characterize the effects of channelrhodopsin-2 (ChR2) transfection in subcortical areas, i.e., the putamen, the external globus pallidus (GPe) and the ventrolateral thalamus (VL) of rhesus monkeys. Lentiviral vectors containing the ChR2 sequence under control of the elongation factor 1α promoter (pLenti-EF1α -hChR2(H134R)-eYFP-WPRE, titer 10^9^ particles/ml) were deposited in GPe, putamen and VL. Four weeks later, a probe combining a conventional electrode and an optic fiber was introduced in the previously injected brain areas. We found light-evoked responses in 31.5% and 32.7% of all recorded neurons in the striatum and thalamus, respectively, but only in 2.5% of recorded GPe neurons. As expected, most responses were time-locked increases in firing, but decreases or mixed responses were also seen, presumably via ChR2-mediated activation of local inhibitory connections. Light and electron microscopic analyses revealed robust expression of ChR2 on the plasma membrane of cell somas, dendrites, spines and terminals in the striatum and VL. This study demonstrates that optogenetic experiments targeting the striatum and basal ganglia-related thalamic nuclei can be successfully achieved in monkeys. Our results indicate important differences of the type and magnitude of responses in each structure. Experimental conditions such as the vector used, the number and rate of injections, or the light stimulation conditions have to be optimized for each structure studied.

## Introduction

Optogenetics have become a mainstream technique to modulate neuronal activity both *in vivo* and *in vitro* [1]. This technology, based on the delivery and expression of light-sensitive ion channel proteins (opsins) in specific neuronal populations, permits stimulation or inhibition of neurons with high temporal and spatial resolution [2], [3]. Optogenetic methods are now frequently employed to study the rodent brain [1], but the use of these techniques in non-human primates has lagged behind, and remains limited to studies of the cerebral cortex [4], [5].

We here report our experience with the use of optogenetic tools in the primate basal ganglia and related thalamic nuclei. These areas are involved in motor planning and execution, as well as non-motor functions, such as the learning of habits and procedures [6], [7], and are the targets of many neurodegenerative disorders, including Parkinson’s and Huntington’s diseases [8]. The basal ganglia networks consists of tightly interconnected subcortical structures including the striatum, the external and internal segments of the globus pallidus (GPe and GPi, respectively), the subthalamic nucleus and the substantia nigra [9]. The motor circuit of the basal ganglia directs its output through the GPi and portions of the pars reticulata of the substantia nigra to the anterior portion of the ventrolateral thalamic nucleus (VLa) [10], [11] Because of the reciprocal nature of these circuits and the complex trajectory of fibers of passage that pierce through the various basal ganglia nuclei, the interpretation of the results of conventional electrical stimulation methods is often complicated. The value of using the far more specific optogenetic techniques to study the physiology of the basal ganglia is well established in rodent studies [12], [13], [14], [15], [16], [17].

It is highly desirable to apply these techniques to study the basal ganglia-thalamocortical circuits in non-human primates as well, because the anatomical organization of these primate basal ganglia and thalamus closely resembles that in humans [18], [19], [20]. We, therefore, studied the function and expression pattern of the cation channel, channelrhodopsin-2 (ChR2), in the putamen, GPe and ventrolateral (VL) thalamus of rhesus macaques, with the assumption that light-activation of ChR2 leads to depolarization of cells expressing the channelrhodopsin. Some of the results of this study have been presented in abstract form [21].

**Figure 1:**
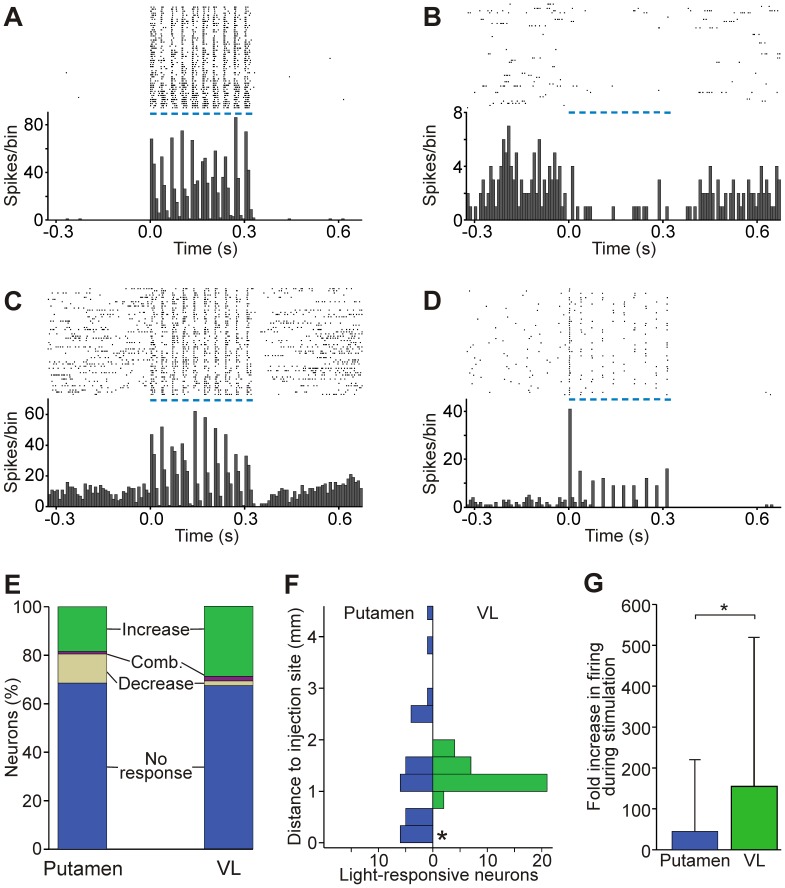
Responses of striatal and thalamic neurons to stimulation with trains of light stimuli. Examples of peri-stimulus time histograms and raster diagrams of responses to light stimulation in the putamen (A, B) and VL (C, D). A, B, D: single units; C: multi-unit. Traces are aligned to the start of light stimulation trains (10 pulses, 20 ms each, 30 Hz), indicated by blue lines. Bin size is 8.125 ms. E: Proportion of responses to light stimulation in each structure. ‘Comb.’ denotes an increase followed by a decrease in firing. F: Distribution of responding cells in relation to the closest injection site. In VL, the closest distance examined was 0.5 mm from the injection site (no optrode tracks at 0 mm, indicated by an asterisk). G: Magnitude of light-evoked increases in firing, expressed as the number of spikes during the stimulation train divided by the number of spikes during the baseline period (means + SD, * p<0.001, Mann-Whitney test).

**Table 1:** Number of neurons responding to light stimulation after lenti-EF1α-ChR2 transfection.

	No Response	Increase	Decrease	Combination*
**Putamen (total)**	63 (68.4)	17 (18.5)	11 (12.0)	1 (1.1)
PAN	5 (50)	1 (10)	4 (40)	0
TAN	26 (76.5)	5 (14.7)	2 (5.9)	1 (2.9)
FSN	9 (75)	1 (8.3)	2 (16.7)	0
**GPe**	78 (97.5)	2 (2.5)	0	0
**Thalamus**	70 (67.3)	30 (28.8)	2 (1.9)	2 (1.9)

Putamen and GPe data were obtained from monkey G, thalamus data were obtained from monkey L. Each column shows the total number and related percentage of cells with specific physiological responses to light stimulation. *, “Combination” responses are increases followed by decreases in firing.

**Figure 2:**
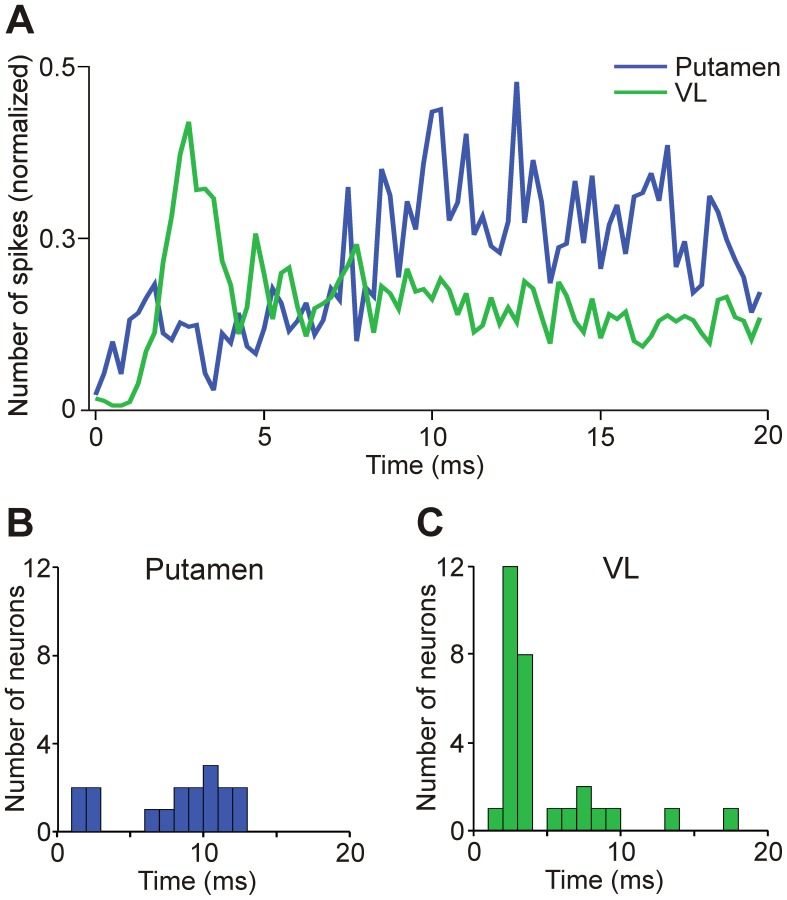
Time course of spiking during a 20 ms light pulse. A: Occurrence of spikes (normalized to the maximal number of spikes in individual cells) during the first 20 ms pulse of each train (50 trials). PSTH bin size is 0.25 ms. B and C: Time to maximal response during 20 ms light pulse for putamen (B) and VL (C) neurons, respectively.

## Methods

### Ethics statement

All experiments were performed in accordance with the National Institutes of Health’s “Guide for the Care and Use of Laboratory Animals” [22], the United States Public Health Service Policy on Humane Care and Use of Laboratory Animals (amended 2002), and were approved by the Institutional Health and Biosafety and Animal Care and Use Committees of Emory University (protocol numbers 232-2009Y and AD08-2530-11).

### Animals and Surgical Procedures

The studies were performed in two male rhesus monkeys (*macaca mulatta*, monkeys G and L, 3 and 4 years, respectively, at the beginning of studies). The animals were raised in the breeding colony at the Yerkes National Primate Research Center and were pair-housed with other monkeys for most of the study, with free access to food and water. At the beginning of the experiments, the monkeys were acclimated to the laboratory, and trained to permit handling by the experimenter and to sit in a primate chair, using positive reinforcement techniques [23].

The animals were surgically prepared for the chronic recording experiments using aseptic conditions and isoflurane anesthesia (1–3%). Stainless steel cylindrical recording chambers (16 mm i.d., Crist Instrument Co., Hagerstown, MD) were affixed to the skull over trephine holes. Using stereotaxic coordinates [24], the chambers were targeted to the putamen/pallidum and VL regions, with a 36° angle from the vertical in the coronal plane. The chambers were fixed to the skull using dental acrylic and stainless steel screws. Metal bolts (Crist Instrument) for head fixation were embedded in the acrylic. After surgery, the animals received prophylactic antibiotic treatment, and were allowed to recover for at least 1 week before the start of electrophysiological and injection procedures. The recording chambers were routinely cleaned at least 3 times every week for the duration of the studies.

### Electrophysiological Recordings

All experimental sessions were conducted with the monkeys awake and seated in a primate chair, with only the head restrained. A tungsten microelectrode (Z = 0.5−1.0 MΩ at 1 kHz; FHC, Bowdoinham, ME) was advanced into the brain with a microdrive (NAN Instruments, Israel), via a 21-gauge guide cannula to pierce the dura mater at the penetration site. Extracellular neuronal electrical signals were amplified (DAM-80; WPI, Sarasota, FL) and filtered in the 400–6,000 Hz range (Krohn-Hite, Brockton, MA) to examine single cell spiking. The filtered signals were monitored with an oscilloscope (DL1540; Yokogawa, Tokyo, Japan) and speakers connected to an audio amplifier. Low frequency components of the electrical brain signals (1–200 Hz) were extracted in parallel (MDA-2; BAK Electronics, Mount Airy, MD). All signals were sampled at 25 ks/s to computer disk (Power1401 and Spike2 software; CED, Cambridge, UK).

During electrophysiological mapping, brain structures were identified according to stereotaxic location, depth in the dorso-ventral plane, relationship to the internal capsule or other landmark structures, and by the characteristic spontaneous neuronal activity in each nucleus. In the putamen, most neurons show very low levels of spontaneous activity, although some (interneurons) are tonically active at higher firing rates [25], [26]. Neurons in the GPe are easily identifiable by their characteristic high frequency discharge, interspersed with pauses [27], [28]. Neurons in the VL can be identified by their irregular activity, and frequent bursting [29].

### Lentiviral Vectors and Injections

We used lentiviral vectors containing the ChR2 sequence under control of the elongation factor 1α promoter, fused to the gene for enhanced yellow fluorescent protein (pLenti-EF1α-hChR2(H134R)-eYFP-WPRE, sequence available at http://www.everyvector.com/sequences/show_public/2501, hereafter called lenti-EF1α-ChR2). The plasmids were provided by Dr. Karl Deisseroth (Stanford University). The viral solutions (10^9^ particles/ml) were prepared by the Gene Therapy Center at the University of North Carolina at Chapel Hill (NC). The vectors were pseudotyped with vesicular stomatitis virus G glycoprotein (VSV-G). For further details regarding the production of viral vectors see ref. [30].

After the electrophysiological mapping, small volumes of the lentiviral vector solution were injected in the GPe, putamen or VL. The injections were guided electrophysiologically, using custom-made systems that consisted of fused silica microinjection tubes glued to conventional tungsten microelectrodes [31], [32], [33]. Monkey G received multiple injections along the medio-lateral and anterio-posterior extent of the sensorimotor GPe and putamen. We injected a total of 16 µl at 9 injection sites in GPe (1.5–2 µl per site) and 20 µl at 10 sites in the putamen (2 µl per site). The injections were delivered at 0.15 µl/min, and the injection system left in place for 10 min after the end of individual injections before withdrawing it from the brain. In monkey L, a single injection of 20 µl was placed in VL, using an incremental ‘convection-enhanced’ injection scheme (0.1, 0.2, 0.5 0.8 µl/min, 10 min each, followed by 1 µl/min for 4 min, modified from the procedure in ref. [34]). As in the other animal, the injection system was left in place for 10 min after the end of the injection before withdrawing it from the brain.

### Photostimulation

To achieve light stimulation of neurons in the vicinity of the sites of electrophysiologic recordings, we used custom-built electrode/optical fiber combinations (‘optrodes’ [35], [36]), which were manufactured by gluing a tungsten microelectrode (0.25 mm shaft diameter, FHC) alongside a polished optical fiber, so that the tip of the electrode protruded approximately 300 µm from the tip of the fiber. Most experiments were done using 200 µm diameter fibers (BFH37-200, 0.37 NA; Thorlabs, Newton, NJ). As noted below, in some experiments fibers with a diameter of 105 µm (AFS105/125Y, 0.22 NA; Thorlabs) were used instead. All fibers were 0.7 – 1 m long.

The optical fiber was coupled to a blue laser (473 nm, 25 mW, Crystalaser, Reno, NV), and the electrode connected to the extracellular recording set up described above. Prior to optrode insertion into the brain, the light output of the fiber was measured with a power meter (PM100USB with a S120C sensor; Thorlabs), and adjusted to a value of 127–270 mW/mm^2^ (corresponding to a laser output of 4–8 mW). The timing of the laser output was controlled via computer-controlled TTL signals, generated with 10 µs precision by the Power1401 unit (CED).

Testing for light-evoked responses started four weeks after the injections. Optrodes were introduced into the brain using the microdrive, and lowered into the structures that were previously transfected with the viral vectors (GPe, putamen, or VL). Based on previous reports [4], [5], we assumed that the highest density of ChR2-expressing neurons would be closest to the injection sites. Optrode penetrations were therefore initially done at the same coordinates as the injections (for putamen and GPe) or 0.5 mm away (VL), with subsequent optrode tracks separated by 1 mm increments in the medio-lateral or antero-posterior directions, until light-responsive units were no longer found.

Once in the nucleus of interest, every single unit (or multi-unit, if single unit isolation was not possible) that could be isolated for a sufficient amount of time with a signal/noise ratio of at least 2∶1 was tested for light responsiveness by applying trains of light pulses (20 ms/pulse, 10 pulses, delivered at 30 Hz, using randomized inter-train intervals of 1–2 seconds). In addition, the effects of single pulses of various durations (20, 10, 5, 2.5, 1.2, 0.6, 0.3, or 0.1 ms) or of pulse trains of a lower pulse frequency (10 Hz) were explored in some experiments (as indicated in Results). On-line spike sorting was used to evaluate light responses during the ongoing experiment (using a waveform matching algorithm, Spike2) and peri-stimulus time histograms (PSTHs) were constructed centered on the start of individual light pulses or trains of light pulses. The optrode was advanced to a new location, if no clear response was observed after 30 trials.

### Data Analysis

All data records were analyzed with off-line spike (re-)sorting, followed by principal component analysis and analysis of inter-spike interval (ISI) distribution histograms to verify the quality of the spike sorting (Spike2). Only neuronal records in which less than 1% of ISIs was shorter than 2 ms were accepted as representing single cells. As indicated in the Results, multi-unit recordings were also included in some sections of the analysis.

#### Classification of striatal and thalamic neurons

As described in previous studies [37], [38], [39], [40], striatal cells can be divided into three main electrophysiologic classes, based on their spontaneous activity: phasically active neurons (PANs), tonically active neurons (TANs) and fast spiking neurons (FSNs), likely corresponding to medium-spiny projection neurons, cholinergic interneurons and parvalbumin-positive GABAergic interneurons, respectively [41]. In this study, we recorded neurons for at least 120 s prior to any light stimulation, and used the firing rates and coefficient of variation (CV) during this epoch for classification [40]. Striatal units were classified as PANs if the firing rate was ≤2.5 spikes/s, and the CV ≥1; as TANs if the firing rate ranged between 2 and 12 spikes/s, with a CV <1, and as FSNs, if the firing rate was >12 spikes/s. Cells that did not fulfill these criteria remained “unclassified”.

In VL, two populations of neurons can be distinguished based on their phenotype, axonal projection and morphology: most are large glutamatergic projection neurons (diameter of soma, 70–400 µm), while smaller sized neurons (soma diameter less than 10 µm) likely represent GABAergic interneurons (around 30% of all neurons) [11]. Although there are no clear electrophysiological criteria to distinguish these cell types, it is likely that most of the recorded neurons in our study were projection neurons, because of the much larger soma size and greater prevalence.

#### Analysis of responses to light stimulation

Following off-line sorting, we constructed PSTHs, aligned to the start of stimulation trains for experiments in which stimulation consisted of 10 pulses of 20 ms delivered at 30 Hz (40 bins per train). Excitatory and inhibitory responses were defined, respectively, as increases or decreases for at least two consecutive bins above or below 2 SD from the neuron’s firing rate during the pre-stimulation portion of the PSTH (i.e., 40 bins before the start of stimulation trains). The PSTHs were used to calculate the magnitude of the light-evoked responses, dividing the total number of events (spikes) generated during the train of light pulses by the total number of events during the pre-train period. Differences in magnitude of responses between putamen and VL neurons were evaluated using a non-parametric Mann-Whitney test (IBM SPSS v20; IBM Corporation Armonk, New York).

We analyzed the time course of the light-induced responses to individual 20 ms light pulses in all neurons that increased firing during light activation. For this, PSTHs were constructed, aligned to the first pulse of each 10-pulse train. Only the first pulse of each train was used in this analysis, as some neurons showed decrementing responses to subsequent pulses in the train (see Results). The time between the start of the illumination and the bin with the largest number of spikes was measured (latency to maximal effect). For an analysis of the time course of spiking responses to the light stimulation across cells, the event number within each bin was normalized to the maximal effect seen in the individual neuron, before collapsing the data across multiple neurons.

In experiments evaluating the effects of altering the length of individual light pulses, PSTHs were constructed aligned to the onset of the pulse. We then determined the magnitude of the stimulation effect as the number of spikes during the 20 ms period after the start of the pulse (regardless of the length of the pulse) divided by the number of spikes in the 20 ms immediately preceding the start of the light pulse. The 20 ms time window was chosen based on the longest single pulse tested.

**Figure 3:**
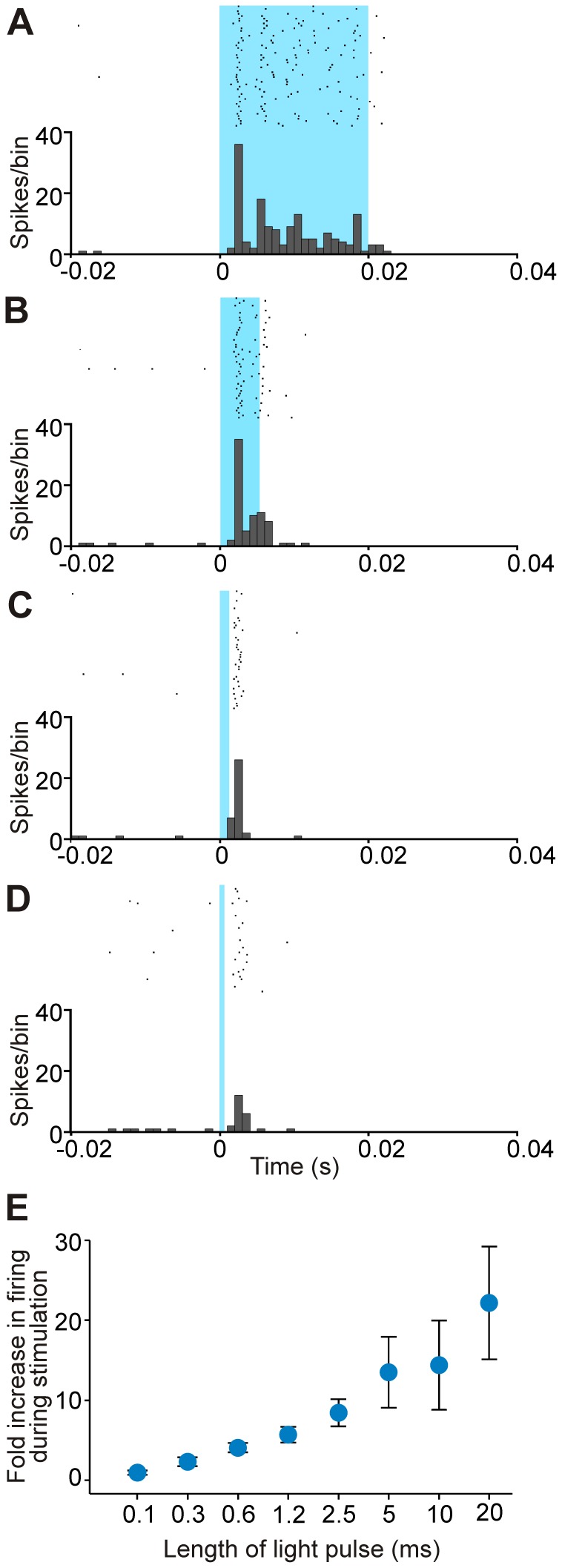
Relationship between light-evoked responses and duration of light pulses. A-D: Examples of peristimulus time histograms and raster diagrams of the response of the same VL neuron to light pulses of decreasing duration. Shaded area indicates length of pulse (20, 5, 1.2 and 0.6 ms, from A to D; 50 trials per histogram, bin size is 1 ms). E: Summary of light-evoked responses to different lengths of light pulses, based on analysis of responses of 11 VL units per data point, except 0.3 ms (n = 9) and 0.1 ms (n = 4). Data points are means ± SEM.

**Figure 4:**
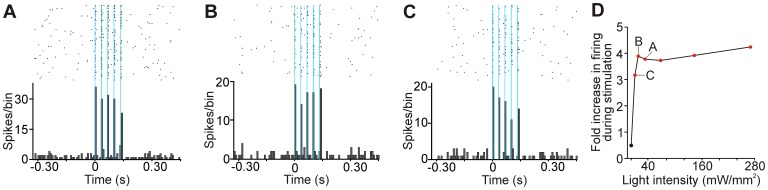
Relationship between light-evoked responses and light intensity. A-C: Examples of peristimulus time histograms and raster diagrams of responses of a VL neuron to trains of light pulses (5 pulses, 1.25 ms each, 30 Hz; bin size is 8.125 ms) of decreasing light intensity (32, 16 and 8 mW/mm^2^, from A to C). Blue shading indicates light pulses. D: Light-evoked responses as light intensity is altered in one VL neuron. The examples in A, B and C are indicated. Red dots indicate experiments in which a significant increase in firing was observed during light stimulation.

**Figure 5:**
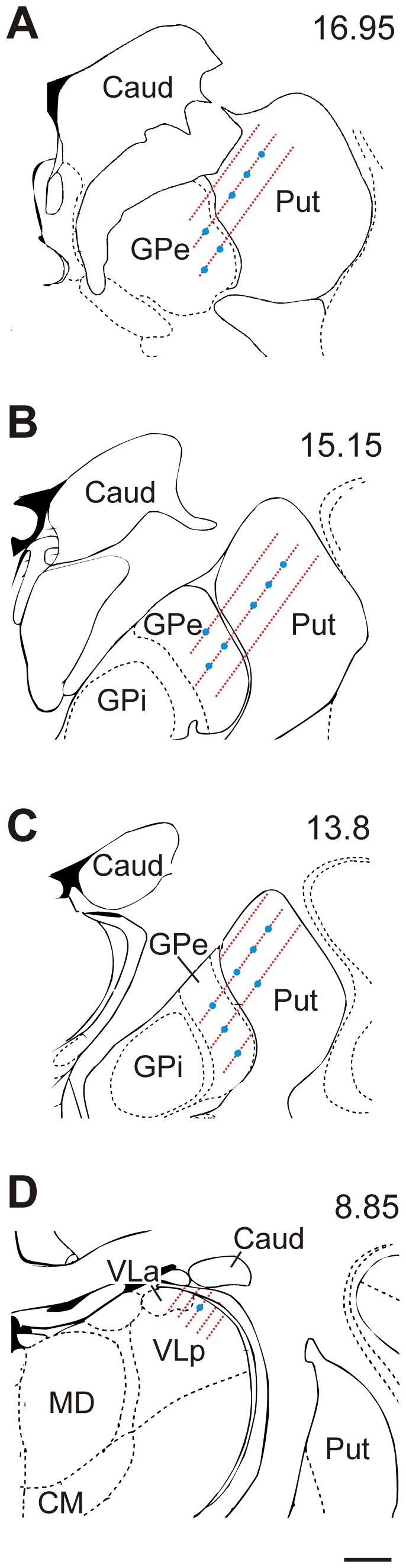
Approximate sites of lenti-ChR2 viral injections and light-stimulation/recording sites. Panels A to C from monkey G, panel D from monkey L. The blue dots indicate approximate sites of viral injections, based on injection tracks and maximal labeling of eYFP. The red lines indicate sites where neuronal recordings coupled to light stimulation were done. The approximate interaural level of these coronal planes is indicated at the top right of each figure, according to ref [24]. Caud: caudate; CM: centromedial nucleus of the thalamus; GPe: Globus pallidus external segment; GPi: globus pallidus internal segment; MD: Mediodorsal nucleus of the thalamus; Put: Putamen; VLa and VLp: anterior and posterior portions of the ventrolateral thalamus, respectively. Scale bar: 2 mm.

### Anatomical Studies

#### Perfusion and initial tissue processing

At the end of the experiment, the animals were deeply anesthetized with an overdose of pentobarbital (100 mg/kg, i.v.), and perfused transcardially with cold oxygenated Ringer’s solution, followed by 4% paraformaldehyde and 0.1% glutaraldehyde in phosphate buffer (PB, 0.2 M, pH 7.4). After removing the brain from the skull, frontal sections (60 µm) were obtained with a vibratome, collected in cold phosphate buffered saline (PBS, 0.01 M, pH 7.4), and stored at −20°C in an anti-freeze solution (30% ethylene glycol/30% glycerol in PB), until processing for light, confocal or electron microscopy, as described below.

#### Light microscopy immunoperoxidase

To identify the expression of ChR2s in the brain tissue after the viral vector infusions, we used antibodies against GFP, which also detect eYFP (fused to ChR2 in the construct used). We selected sections containing the putamen and/or GPe in monkey G and the VL in monkey L, in which injection tracks were visible. After pretreatment with 10% normal goat serum (NGS) and 1% bovine serum albumin (BSA) in PBS, the sections were incubated for 24 h at room temperature in the primary antibody solution (anti-GFP, made in chicken, 1∶2000, catalog no. 06–896; Millipore, Billerica, MA). To reveal the primary antibodies, the sections were incubated in biotinylated goat anti-chicken IgG (1∶200; Vector Laboratories, Burlingame, CA) for 2 hr, and in avidin-biotin complex (ABC) solution (1∶100; Vectastain standard kit, Vector) for 90 min. The sections were then rinsed in PBS and TRIS buffer (0.05 M, pH 7.6) and placed in a solution containing 0.025% 3-3'-diaminobenzidine tetrahydrochloride (Sigma-Aldrich, St. Louis, MO), 0.01 M Imidazole (Fisher Scientific, Pittsburgh, PA) and 0.006% H_2_O_2_. The reaction was terminated by repeated washes in PBS. All immunoreagents were diluted in PBS containing 1% NGS, 1% BSA and 0.1% Triton X-100. The sections were mounted on gelatin-coated slides, dehydrated in alcohol, immersed in toluene and a coverslip was applied with Cytoseal XYL (Richard-Allan Scientific, Kalamazoo, MI). The slides were digitized with an Aperio Scanscope CS system (Aperio Technologies, Vista, CA). For the double immunofluorescence and electron microscopy studies, we selected sections adjacent to those that showed strong eYFP labeling.

#### Double immunofluorescence

To assess whether the transfection was specific for neuronal elements, we used a double immunofluorescence protocol to label eYFPs associated with ChR2, and antibodies against glial fibrillary acidic protein (GFAP). To evaluate the expression of ChR2 in cholinergic striatal interneurons, we used choline acetyl transferase (ChAT), a specific marker of these cells [42], [43]. After treatment with sodium borohydride (20 min) and repeated rinses with PBS, sections were pre-incubated in 5% normal donkey serum (NDS), 1% bovine serum albumin (BSA) and 0.3% Triton X-100, and incubated overnight at room temperature in a cocktail of anti-GFP (made in rabbit, 1∶5000, catalog no. A-11122; Invitrogen, Grand Island, NY) and anti-GFAP (generated in mouse, 1∶7500, catalog no. MAB360; Millipore) or anti-ChAT (made in goat, 1∶100, catalog no. 50–265; Pro-Sci, Poway, CA). Then, sections were rinsed with PBS and incubated for 60 min in the corresponding secondary antibodies solution (1∶100) as follows: Fluorescein (FITC)-conjugated donkey anti-rabbit IgGs to reveal eYFP, Texas red-conjugated goat anti-mouse IgGs to reveal GFAP, or rhodamine red-X-conjugated donkey anti-goat IgGs to reveal ChAT (all from Jackson Immunoresearch Laboratories, West Grove, PA). The primary and secondary antibodies were diluted in PBS containing 1% NDS and 1% BSA. Sections were washed in PBS and transferred to a solution (pH 5.0) containing 0.4% ammonium acetate and 0.16% cupric sulfate in distilled water for 30 min, and after several rinses in PBS, sections were mounted with Vectashield (Vector). To examine the sections, we used confocal laser scanning microscopy (DM5500B; Leica Mircosystems, Bannockburn, IL) equipped with a CCD camera (Orca R2; Hamamatsu, Bridgewater, NJ). To determine the proportion of ChAT-positive cells that were also eYFP-positive (expressing ChR2), we scanned double immunolabeled tissue, and acquired digital micrographs (63x) of all ChAT-positive cell bodies encountered in areas where the eYFP fluorescence was found. One image of each cell was taken. A total of 131 cells were counted in 5 sections. The digital images were opened using ImageJ [44] and the red or green filters minimized to identify cell bodies that expressed only ChAT (red), only eYFP (green) or both.

### Electron Microscopy

The ultrastructural localization of ChR2s in the viral transfected tissue was analyzed at the electron microscope level, using pre-embedding immunoperoxidase and immunogold methods. Sections to be processed for EM observations were first treated with 1% sodium borohydride, placed in a cryoprotectant solution (PB, 0.05 M, pH 7.4, containing 25% sucrose/10% glycerol) for 20 min, frozen at −80°C for 20 min, thawed and washed in PBS. The immunoperoxidase method is very sensitive, but the diffuse nature of the peroxidase reaction product limits the spatial resolution of the method, and obscures the details of antigen expression in intracellular organelles and membranes. It is, therefore, useful to complement this high sensitivity/low resolution method with the immunogold technique, which is less sensitive, but offers a much higher spatial resolution [45].

Thus, to further extend the localization of ChR2 labeling and demonstrate its association with the plasma membrane of neuronal elements, immunogold processing was carried out in striatal tissue from monkey G. Sections to be processed with immunogold antibodies were immersed in PBS containing 5% non-fat dry milk, and washed in TBS-gelatin buffer (Tris 0.02 M, NaCl 0.15 M and 0.1% cold water fish gelatin). After incubation in the primary antibodies (anti-GFP, 1∶5000; Invitrogen, 24 hr, 4°C), the sections were rinsed in TBS-gelatin and incubated in gold-conjugated anti-rabbit IgGs (1∶100; 1.4 nm particle size, Nanogold; Nanoprobes, Stony Brook, NY) for 2 hr at room temperature. Primary and secondary antibodies were diluted in TBS-gelatin buffer containing 1% dry milk. The sections were then washed with TBS-gelatin and 2% acetate buffer (pH 7.0) and the gold labeling was silver-intensified (HQ silver; Nanoprobes) for 9–13 min at room temperature, in the dark. The process was stopped by several washes in acetate buffer. The tissue was post-fixed in osmium tetroxide (0.5% in 0.1 M PB, pH 7.4), rinsed in PB, and dehydrated by exposure to increasing concentrations of ethanol and propylene oxide. Uranyl acetate (1%) was added to the 70% ethanol solution. Sections that were processed for immunoperoxidase staining received the same treatment as described above for the light microscope study, but Triton X-100 was omitted from all incubations and 1% osmium tetroxide was used. After revealing the GFP antibody labeling with DAB, the sections were washed in PB (0.1 M, pH 7.4), post-fixed in osmium and further processed as described above.

Sections were then embedded in resin (Durcupan ACM; Fluka, Ft. Washington, PA) on microscope slides and placed in a 60°C oven (48 hr). The sections were examined under the light microscope, and blocks of tissue were cut in areas rich in eYFP labeling, and glued onto resin blocks. Ultrathin (60 nm) sections were obtained with an ultramicrotome (Ultracut T2; Leica, Nussloch, Germany) and collected on pioloform-coated copper grids. To enhance contrast, sections were stained with lead citrate (5 min). Tissue was examined with an electron microscope (JEOL model 1011, Peabody, MA) at 20,000–25,000X and micrographs of fields containing immunoreactive elements were acquired using a digital camera (DualView 300W; Gatan, Inc., Pleasanton, CA) controlled by DigitalMicrograph software (version 3.10.1, Gatan).

To quantify the cellular elements expressing ChR2 (eYFP-positive), we examined tissue processed for immunoperoxidase (1270 and 820 µm^2^ of striatal and thalamic tissue respectively were analyzed). The labeled structures were categorized into dendrites, terminals, unmyelinated and myelinated axons, neuronal somata and glial processes based on ultrastructural features [46]. Small diameter elements (<0.5 µm) were classified as unmyelinated axons if they had a circular shape, were part of a larger bundle of axons, did not receive synaptic contacts, or contained synaptic vesicles. Small dendrites had a more irregular shape, often contained mitochondria, and were commonly found in synaptic contact or apposed to axon terminals, while glial processes were thin, often displayed a tortuous shape to fill space between neuronal structures, and occasionally contained glial filaments. Small immunoreactive elements that could not be categorized based on these ultrastructural features were labeled as “unidentified”. Dendritic elements were further categorized as small (<0.5 µm), medium (0.5–1.0 µm) or large (>1.0 µm), according to their cross-sectional diameter. The relative proportion of each category of labeled elements was calculated and expressed as percent of total labeled elements in the areas examined, as previously reported in other studies [31], [47].

**Figure 6:**
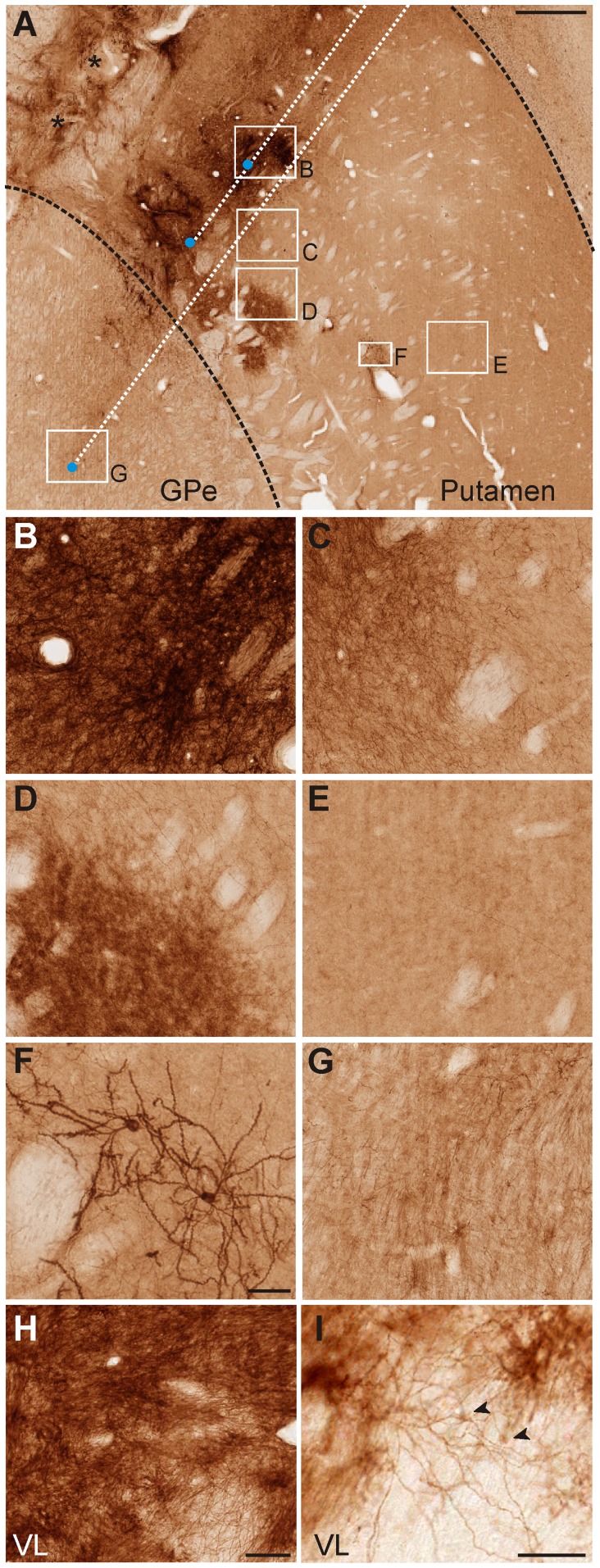
Extent of channel rhodopsin transfection after lentiviral injections in the putamen, GPe and VL. The low magnification image (A) shows injection trajectories (dotted white lines) and approximate sites of injection (blue dots) in the putamen and GPe. Asterisks indicate eYFP-peroxidase labeling produced by injection tracks not shown in this section. The sites at which images B-G were taken are indicated as squares. The images in B-F show examples of eYFP-immunopositive (ChR2) staining at various distances from the injection site in the putamen and GPe. The images in H and I show examples of eYFP peroxidase labeling in the VL, close (H, ∼0.5 mm) and distal (I, >1 mm) to the injection site. Arrowheads in I point to eYFP-positive neuronal cell bodies. Scale bars: A, 1 mm; H, 0.1 mm (applies to B, C, D, E, G); F and I, 50 µm. GPe: external segment of the globus pallidus; VL: ventrolateral nucleus of the thalamus.

**Figure 7:**
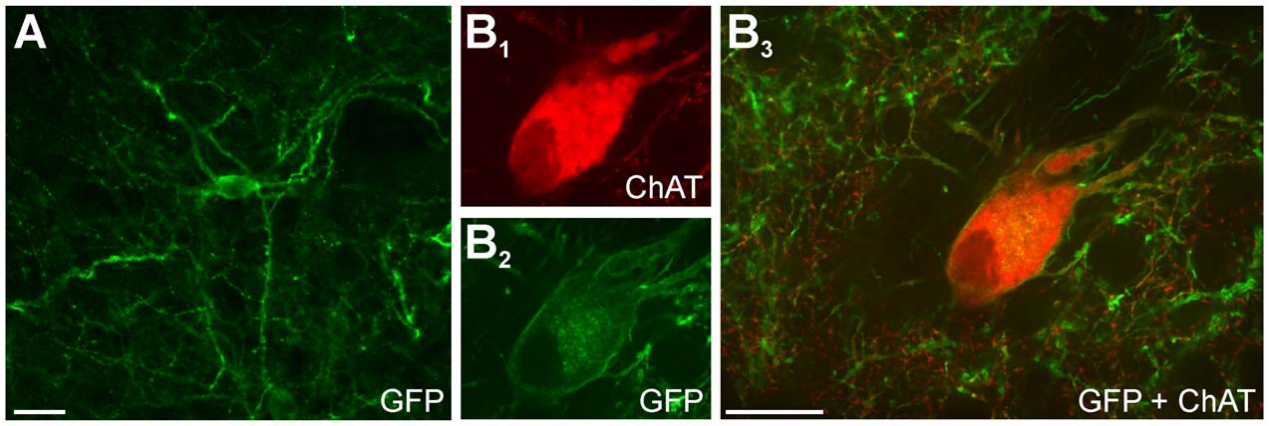
Different subpopulations of striatal cells express channel rhodopsins. A: In striatal sections stained with antibodies against eYFP (FITC as fluorophore), a subpopulation of neurons showed clear staining in the cell body and along the dendritic tree. In separate sections (B), double staining using eYFP (+ FITC) and ChAT (+ Texas Red) revealed that some neurons expressing eYFP were also positive for ChAT. Scale bars: 25 µm (scale bar in B_3_ applies to B_1_ and B_2_).

## Results

### Electrophysiological Studies

We included 92 striatal, 80 GPe cells and 104 thalamic single or multiunit recordings in the analysis. Based on the pre-stimulation firing rate and coefficient of variation (CV, see Methods), striatal single unit recordings were divided into PANs (n = 10), TANs (n = 34), FSNs, (n = 12) and unclassified (n = 36).

### Type and Magnitude of Responses to Light Stimulation

The optrode penetrations were performed in the vicinity of the viral injection sites. We found 29 striatal single units (31.5% of all recorded striatal neurons) and 34 thalamic units (24 single and 10 multi-units, 32.7% of all recordings) with significant changes in firing rate in response to the light stimulation (Fig. 1). Very few GPe cells were affected by the illumination (only 2/80 cells showed light-evoked responses), suggesting that the viral transfection in GPe was not effective, as later confirmed in the histological examination (see below). Control experiments in non-transfected areas never resulted in light-induced changes in activity. As previously reported [4], [48], delivery of light next to the recording electrode evoked a low-frequency electrical artifact (Becquerel effect) in control and lenti-EF1α-ChR2 transfected areas (not shown).

Given that activation of cell membrane expression of ChR2 results in transport of positive charges into the cytoplasm of the expressing cell [49], illumination induced increases in firing in most responding neurons (Fig. 1A, C, D). However, 12% and 1.9% of responding cells in the striatum and thalamus, respectively, showed decreases in firing (Fig. 1B). Also, a small number showed combinations of increases and decreases in firing. A summary of the effects is shown in Fig. 1E. In the putamen, light-stimulated effects (both increases and decreases of firing) were observed in PANs, TANs and FSNs (Table 1). For the subsequent analysis, all striatal cells that responded to the stimulation were pooled together. Most of the light-sensitive neurons were located within 2 mm of the closest injection site (Fig. 1F). The magnitude of responses for cells that showed increases in firing was calculated as the number of events (spikes) during light stimulation normalized to the number of events during the same period of time before the onset of light. Based on this analysis, we found that thalamic cells (as a group) generated significantly more spikes in response to the optical stimulation than striatal neurons (45±177 fold increase in firing [mean ± SD] in the putamen, and 155±365 in VL, p<0.001, Mann-Whitney test, Fig. 1G).

#### Time course of light-evoked responses

When tested with a train of 10 pulses delivered at 30 Hz, about 40% of neurons in the putamen or VL showed a similar magnitude of responses to every pulses in the train (Fig. 1A, C), while in other neurons, the first pulse in the train lead to a larger spiking response than subsequent pulses (Fig. 1D). Four cells in the VL that showed this pattern of decreasing responses with the 10 pulses/30 Hz stimulation parameters were also tested with a 5 pulses/10 Hz regime. Even with the lower frequency of stimulation, the number of evoked spikes was 20–30% higher in response to the first pulse than to the subsequent pulses (not shown).

In most cells, the firing rate changes evoked by light stimulation were restricted to the period of illumination, but 4 neurons in the putamen and 5 in VL showed significant decreases in firing rates after the end of the train of light pulses (Fig. 1D). These reductions in firing lasted 38±35 ms (mean ± SD) in the putamen, and 169±81.6 ms in VL.

We next examined the time course of the light-induced responses to individual 20 ms light pulses (i.e., the first pulse in each pulse train). Striatal neurons generated more spikes during the second half of the 20 ms light pulse, while thalamic cells showed a more rapid and shorter-lasting increase in firing (Fig. 2A). Most striatal neurons reached the maximal effect at around 10 ms into the pulse, while the majority of VL neurons showed the largest response within the first 5 ms of the pulse (Fig. 2B, C).

#### Dependence of light responses on length and intensity of light exposure

In a group of VL neurons that showed increases in firing, we were able to test the effects of different light pulse durations (11 units, Fig. 3). With the exception of one neuron that showed no significant increase in firing with pulses shorter than 5 ms, light pulses as short as 0.6 ms produced significant responses. In a few cells, even shorter pulse durations could be tested. Stimulation with 0.3 ms pulse width sufficed to evoke spiking in 6/9 neurons tested in this manner, while pulses of 0.1 ms duration were ineffective (tested in 4 cells). The responses evoked by pulses of up to 1.2 ms duration started after the end of the pulse (Fig. 3C, D). Wider pulses resulted in an overall larger number of light-evoked spikes, as the neuronal activity increased throughout the light exposure (even though the peak of the responses occurred early in the pulse, as examined above; Fig. 3E).

In one VL neuron, we also examined the minimal light intensity required to elicit light responses, by reducing the laser power, and keeping the same pulse length. The magnitude of responses remained constant as we decreased the laser output from 270 mW/mm^2^ (measured at the tip of the fiber, see Methods) to 16 mW/mm^2^. A significant increase in firing was observed even when using light intensities as low as 8 mW/mm^2^ (Fig. 4).

Finally, we tested the possibility of using optrodes built with smaller diameter optical fibers (in which the light intensity at the tip is smaller). In cells tested using 105 µm diameter fibers (instead of the 200 µm diameter of the fibers used for all other experiments), we observed increases in firing that were 1.8 to 34 times the baseline firing. Thus, thin optical fibers can be successfully used in such experiments.

**Figure 8:**
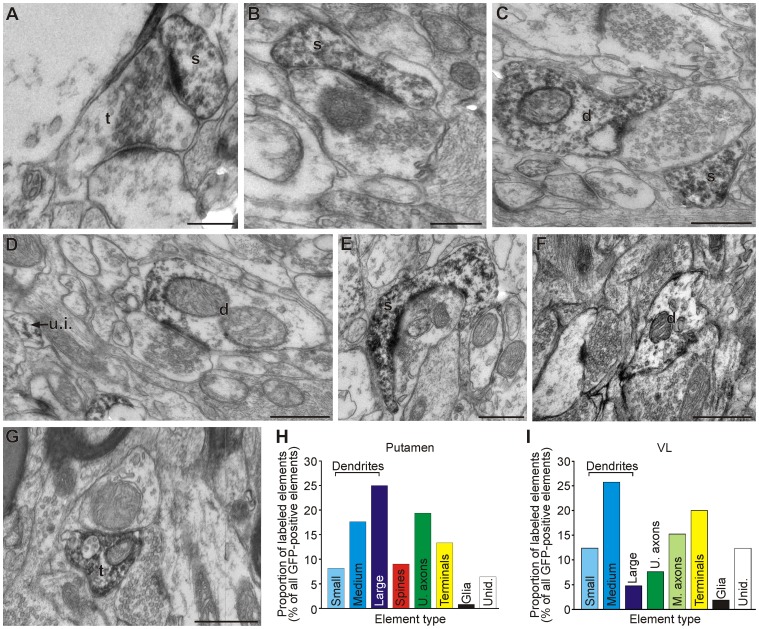
Channel rhodopsins are expressed in pre- and post-synaptic neuronal elements. A-E: Examples of eYFP-positive (ChR2 expressing) neuronal elements in the striatum (A-E, from monkey G) and VL (F, G, from monkey L). H and I: Proportion of immunolabeled elements in Putamen and VL. Scale bars: A-B, 0.25 µm; C-G: 0.5 µm. Abbreviations: d, dendrite; M. axons, myelinated axons; s, spine; U. axons, unmyelinated axons; t, terminal; u.i. or Unid., unidentified elements.

**Figure 9:**
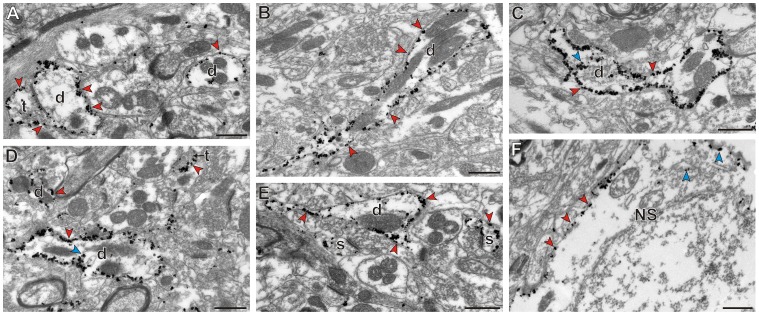
Channelrhodopsins are expressed almost exclusively at the neuronal plasma membrane. A-F: Examples of immunogold staining (indicating ChR2 expression) in the putamen. Arrowheads point at plasma membrane-bound (red arrows) or intracellular (blue arrows) gold particles. Abbreviations: d, dendrite; s, spine; t, terminal; NS, Neuronal soma. Scale bars: 0.5 µm (applies to all panels).

### Anatomical Studies

The transfection of lenti-ChR2 was assessed using antibodies against eYFP. The location of injections was confirmed in the putamen and GPe for monkey G and in the ventrolateral portion of the motor thalamus in monkey L (Fig. 5).

In monkey G, strong peroxidase labeling (indicating ChR2-expressing areas) was found in the vicinity of the putamen injection sites (Fig. 6A). Expression level gradually decreased with increasing distance from the center of the injection, until it reached a relatively abrupt limit, 1–2 mm away from the injection sites, beyond which little staining was found, except for a few isolated neurons (Fig. 6B-F). In the core of the injection site, the dense neuropil staining made it difficult to differentiate individual cell bodies or dendrites (Fig. 6B). However, the cell bodies that could be unequivocally visualized with this staining method had a neuronal morphology. In double labeling experiments, eYFP-positive elements were segregated from glial profiles, revealed with anti-GFAP antibodies (Fig. S1), indicating that the ChR2 expression was confined to neuronal elements. These observations were confirmed in the ultrastructural analysis (see below). Some of the opsin-expressing neurons resembled medium spiny projection neurons, based on the cell body size and the presence of spines covering the dendritic processes (Fig. 6F, 7A). As expected based on the poor responses to light stimulation, lentiviral injections in GPe resulted only in weak opsin expression in some neuropil processes, without any significant neuronal cell body labeling (Fig. 6A, G).

In monkey L, the single viral injection resulted in transfection of the dorsal part of the ventrolateral motor thalamus, including both basal ganglia and cerebellar-receiving areas (VLa and the posterior portion of the VL (VLp) respectively, Fig. 5D). Similar to the observations in the putamen, strong neuropil labeling in which specific neuronal elements were difficult to differentiate at the light microscopic level was found close to the VL injection site (Fig. 6H), but when thalamic areas located more than 1 mm from the core of the injection site were examined, individual dendritic processes and neuronal cell bodies could be identified (Fig 6I).

We expected that opsin expression would not be restricted to specific neuronal subtypes (due to the use of the ubiquitous promoter EF1α in the viral vector). Indeed, our electrophysiological results showed light activation in neurons that were classified as striatal projection neurons and interneurons. To further confirm this heterogeneous opsin expression across different striatal neurons, we used antibodies against choline acetyltransferase (ChAT), a reliable marker of cholinergic interneurons (see Methods), to determine the proportion of these cells that expressed opsin in the close vicinity of the injection sites. Confocal microscope observations revealed striatal areas that were double stained with eYFP antibodies (to reveal opsin expression), and ChAT antibodies (to label cholinergic interneurons; Fig. 7). We determined if ChAT-positive cell bodies expressed eYFP labeling in a total putamen area of 1.5 mm^2^ (5 sections). Of the 110 ChAT-positive neurons examined, 39 (35%) expressed eYFP immunoreactivity (Fig 7B), a proportion that is in good agreement with the overall percentage of TANs (presumably cholinergic interneurons) that responded to light-stimulation in the electrophysiology experiments (Table 1).

Observations at the light and confocal microscope levels revealed strong dendritic expression of ChR2 in both the striatum and VL, and spine-like processes in the striatum, as well as other elements in the neuropil. The ultrastructural identity of ChR2-positive neuronal compartments was examined at the electron microscope level, with blocks of tissue prepared from striatal and thalamic sections close to the injection sites.

The EM observations showed that exposure to lenti-EF1α-ChR2 did not alter the ultrastructural morphology and integrity of the transfected areas (Figs. 8 and 9). As shown in fig. 8, ChR2 expression was frequently found in postsynaptic elements. Of all labeled elements, 51 and 43% were identified as dendrites in the putamen and thalamus, respectively. In the putamen, 9% of the labeled elements were dendritic spines. Albeit to a smaller extent, some presynaptic elements were also found to be labeled. Unmyelinated axons accounted for 19 and 8% of labeled elements in the putamen and VL, respectively, while 13% and 20% of labeled elements were terminals, in the putamen and VL, respectively. In the VL, 15% of labeled elements were myelinated axons. In both structures, immunolabeling was observed even in small dendrites (diameter <0.5 µm) and also in striatal spines, indicating that the expression of opsins was not confined to large (presumably more proximal) dendrites (diameter >1 µm). In both the putamen and VL, less than 2% of labeled elements were identified as glial processes.

Immunogold labeling was used to define the location of the ChR2 expression in relation to the plasma membrane in labeled structures. The gold particles were localized almost exclusively along the plasma membrane of eYFP-positive pre- and post-synaptic neuronal elements (Fig. 9). Only a few particles were found in the intracellular compartment, where they seemed to be associated with unidentified organelles (Fig. 9F).

## Discussion

Striatal and thalamic neurons in rhesus macaques were successfully transfected with lenti-EF1α-ChR2. In contrast, poor opsin expression or light-evoked responses were found in GPe neurons using this viral vector. As reported previously [4], the animals tolerated the lentiviral injections without ill effects. Our results demonstrate that the optogenetic technique can be used to control neuron activity in basal ganglia and thalamic regions of monkeys *in vivo*, opening the door to future optogenetic studies of the connectivity and functions of the basal ganglia in normal and pathological conditions in these animals.

We found substantial differences in the proportion of neurons that expressed ChR2s after lentiviral transfection, as well as the type and magnitude of responses. Thus, the methods that worked well in the striatum and thalamus were unsuccessful in the globus pallidus, thereby indicating, as suggested by Yizhar et al. [50], that the viral construct, injection parameters and light stimulation conditions need to be optimized for each structure to be studied.

### Specificity and Reliability of the Lenti-EF1α Vehicle

We used lentiviral vectors and the EF1α promoter to achieve neuronal opsin expression. Lentiviral vectors were found to be safe and effective when delivered in basal ganglia regions in previous macaque studies (e.g. [51], [52] ). However, injections of lentivirus solutions tend to lead to relatively limited expression of their cargo in the tissue [50] (see below). Other vectors, for instance, adenovirus associated virus, serotype 5 (AAV-5), may lead to more widespread transfection, as has been shown in studies of viral expression in the monkey cerebral cortex [5].

To maximize our chances of finding opsin expression, we used EF1α a ubiquitous promoter [53], that demonstrates mainly neuronal preference in the brain when used in combination with lentiviral vectors [54]. We confirmed that this promoter drives construct expression in a variety of neurons, including projection neurons and interneurons in the striatum and the thalamus. This conclusion is based on electrophysiological features of neurons that responded to light, and was confirmed with histological analysis of striatal tissue. However, lenti-EF1α did not simply drive opsin expression in all neurons in the vicinity of the injection site, as GPe cells were obviously not effectively transfected after the virus solution injections. This further confirms that the efficacy of a promoter-vector construct observed in one brain region may not be generalized to other nuclei, as previously noted [50]. It is not clear whether the vectors were unable to transfect pallidal cells due to different sensitivity of GPe neurons to transgene expression, or whether the lentiviral vectors transfected these neurons, but the opsin expression was not achieved. Other studies have used lentiviral vectors to interfere with the mRNA expression of corticotropin-releasing factor receptors in pallidal cells in mice [55], but there are no previous reports of lentiviral injections in the monkey GPe. Further testing is needed to identify a viral vector-promoter combination that improves opsin expression in nonhuman primate pallidal neurons in. Pan-neuronal promoters (e. g., synapsin) or neuron-specific promoters could be used in future experiments, as they become available, to examine the effects of activating specific neuronal populations within the subcortical structures under study.

### Limited Diffusion of Lentiviral Solution and Proportion of Cells Transfected

The histological analysis demonstrated that diffusion of lentiviral solutions was limited to a few millimeters from the injection tracks in the striatum and thalamus. Accordingly, electrophysiological responses to light stimulation were confined to be within 1 mm of the injection sites. This relatively restricted area of opsin expression is similar to that found in a previous report by Han et al. who showed lentivirus-based transfection to be limited to a 1.4±0.5 mm diameter sphere of tissue in the monkey frontal cortex [4]. Such limited expression can be advantageous when the technique is applied to small brain areas, like the subthalamic nucleus, but there may be a need for using multiple injections or much larger injection volumes when attempting to cover larger structures, such as the striatum. The use of other viral vectors may also be advantageous (see, e.g., ref. [5]).

We found that the proportion of light-sensitive neurons in the putamen (31.5%) and VL (32.7%) after ChR2 transfection is lower than the proportion of similar neurons previously reported in the monkey cerebral cortex. Thus, Han et al. reported that all cells responded to light stimulation in the limited area transfected with lenti-EF1α-ChR2 [56], while Diester et al. found a responder rate of 45–50% of cells after cortical AAV5-ChR2 transfection [5]. Although the different proportions of light-sensitive cells could be the result of different sampling and recording strategies, it is noteworthy that percentages similar to ours were found in a previous study of the mouse striatum [57], albeit achieved with a different viral-transfection strategy.

### Light Modulation of Neuronal Activity and Differences among Structures

As expected, given the nature of the excitatory opsin, most electrophysiological responses of striatal and thalamic neurons were increases in firing rates during the delivery of light pulses. Most neurons showed a stronger response to the first than to subsequent pulses during a stimulation train. It is possible that this outcome results from the inability of ChR2 to follow relatively high frequencies of stimulation [58], [59]. However, even lower frequencies of stimulation (10 Hz) did not restore full responses to each pulse in the train, suggesting that the decreasing responses could be a consequence of the cell-endogenous properties, or of prominent interactions with the local circuitry. For instance, neurons in the vicinity of the recorded neuron may be stimulated and may act to partially inhibit the responses of the recorded unit.

While most VL cells increased their firing during light stimulation, approximately 40% of light-responding neurons in the striatum showed reductions in firing rates. These inhibitory responses are likely to result from indirect effects mediated through light stimulation of neighboring GABAergic interneurons or inhibitory axon collaterals of projection neurons within the striatum [41], [60]. Similarly, Han et al. [4] found suppression of neuronal activity after light stimulation in a ChR2-expressing area in the monkey cerebral cortex. Thus, despite being a cation channel, activation of ChR2s can elicit neuronal responses of opposing polarities within the same brain nucleus. Furthermore, even within the group of neurons that increase firing during illumination, we noted heterogeneity in the magnitude of responses and in the reliability of response from pulse to pulse. In addition, some of the light-responding units showed significant changes in firing rates (mostly inhibitions) after the end of the illumination period. An important lesson from these experiments is, thus, that the downstream effects of light stimulation of ChR2-transfected nuclei may not match expectations based solely on the physiological effects of ChR2 activation in the transfected neurons, but also rely on the complex local network of direct or indirect inhibitory interactions within the stimulated nucleus. This is not a shortcoming of the optogenetic method, but simply reflects the fact that the biological system is more complex than the models tested with it.

The magnitude of excitation achieved with ChR2 activation appears to vary between brain regions. In our studies, light-excited VL cells as a group, had an average 155% increase in firing compared to 45% in striatal cells. In the monkey frontal cortex, ChR2-transfected cells increased their firing rate transiently by up to 750% when light-stimulated [4]. The differences in magnitude of firing increases in response to light among different population of ChR2 expressing cells may be the result of the endogenous firing capability of neurons in each region, combined with differences in amount and pattern of ChR2 expression.

The temporal response to light pulses of 20 ms duration differed between VL and putamen neurons. VL neurons reached their maximal response rapidly (in less than 5 ms), while the increased firing of striatal cells ramped up more slowly, and was more prominent after 10 ms. However, regardless of the timing of the peak response, light-induced spikes occurred during the entire duration of illumination in both structures (at least up to pulses duration of 20 ms). In the monkey frontal cortex, 200 ms light pulses increased the firing rate of ChR2-expressing neurons throughout the duration of the pulse, although the strongest response was observed during the first 20 ms of illumination [4].

On the other end of the temporal spectrum, we found that pulses as short as 0.6 ms elicited significant spiking responses, likely as a direct consequence of the rapid activation and relatively slow deactivation kinetics of the ChR2-H134R variant (τ_on_  = 1 ms; τ_off_  = 20 ms [61]). In other studies, pulses as short as 0.3 ms were shown to elicit light responses in ChR2-transfected neurons in the monkey cortex [5]. For *in *vivo experiments, the use of shorter pulses can be advantageous to reduce the total amount of light delivered in the tissue, and to prevent potential thermal tissue damage [48], [50].

Studies by others have shown that light power intensities of 1–5 mW/mm^2^ are sufficient to activate ChR2 *in vitro* [62], and previous studies in monkey cortex demonstrated that 3 mW/mm^2^ may suffice in some *in vivo* situations [5]. In good agreement with these results, we found that a minimal light intensity of 8 mW/mm^2^ was required to elicit significant spiking in VL neurons. Considering these light requirements, it seems feasible, as we later confirmed, to use thin (<200 µm) optical fibers to illuminate the neurons in close vicinity to the recording electrode, and thus, to minimize the tissue damage by repeated probe insertions in the brain.

### Cellular and Subcellular Pattern of Expression of Channelrhodopsins

We used light and electron microscopy to evaluate the ultrastructural integrity of ChR2-transfected tissue and to describe the expression of ChR2. These studies showed strong expression of opsins along the dendritic arbors of striatal and thalamic cells, including distal dendrites and spines, suggesting that ChR2 was transported through large portions of the dendritic tree. Ultrastructurally, labeling was found in postsynaptic structures (i.e. cell bodies, dendrites, spines), and presynaptic elements (terminals and unmyelinated axons). The ChR2-positive presynaptic elements probably originated from striatal or thalamic cells that were transfected with the lentivirus in which the opsin was transported anterogradely to terminals within the structure. Thus, our ultrastructural observations suggest that ChR2s on dendrites and cell bodies are primarily responsible for the modulation of firing rates during light stimulation, but that responses to light stimulation may also be the result of presynaptic regulation of transmitter release from ChR2-expressing terminals in the vicinity or in contact with the recorded neurons.

Our light microscopic observations, showed only faint or no labeling in those brain areas that receive afferents from the striatum (GPe, GPi, and substantia nigra) or VL (motor cortical areas), indicating that the extent of anterograde transport to projection targets was negligible. We also did not find evidence of retrograde transport of the opsins in areas known to project to the striatum (such as the substantia nigra, cerebral cortex and thalamus) or the VL (such as GPi, but cerebellar regions were not examined), in agreement with the reported lack of retrograde labeling after lenti-EF1α-ChR2 in the monkey frontal cortex [4].

The preferential targeting of ChR2s to the plasma membrane of transfected cells has been previously shown with fluorescence microscopy [4], [35], [61], [62]. Our electron microscope data extend these observations showing that most channelrhodopsin molecules (as assessed by immunogold labeling) are bound to the plasma membrane of labeled neuronal elements, indicating that the opsins are placed in optimal locations to be activated by light.

### Conclusion

We found that the use of optogenetic tools in nonhuman primate subcortical structures is feasible, and that these techniques can be applied to studies of the circuitry and functions of basal ganglia and related thalamic nuclei. However, our data also suggest several important aspects of the use of optogenetic techniques that need to be considered in the interpretation and design of future monkey studies: (1) at most one third of the neuronal population in the thalamus and striatum was light-sensitive after lenti- EF1α-ChR2 transfection, (2) the net physiological responses to ChR2 activation is not solely the result of direct ChR2-induced excitatory influences upon the stimulated neurons, but may also involve indirect effects, most likely mediated through local inhibitory network activation, and (3) the extent of neuronal transfection and light stimulation effects upon transfected neurons are highly region-specific. Several challenges remain to be addressed to further expand the use of optogenetics in basal ganglia and related structures in non-human primates *in vivo*, including the availability of promoters for specific cell types, the development of strategies to enlarge the viral-transfected areas, the need to increase the proportion of light-responsive neurons, and the need to achieve illumination of larger areas. Further development of these techniques for use in monkeys is clearly worthwhile as they may help us to explore normal functions of the basal ganglia and other structures, and to examine pathologic activity patterns in disease models that closely resemble human disorders.

## Supporting Information

Figure S1 **Glial elements do not express channelrhodopsins after lenti-ChR2-αEF1 transfection.** Confocal fluorescence micrographs in the putamen showing eYFP/ChR2-positive (A) and GFAP-positive (glial) elements (B). ChR2-positive elements do not express GFAP, as shown in the merged imaged in (C). Scale bar: 20 µm.
(TIF)
