# Supplementary figures and images for: In Vivo Optogenetic Control of Striatal and Thalamic Neurons in Non-Human Primates
Source: PLoS One. 2012 Nov 30;7(11):e50808. doi: 10.1371/journal.pone.0050808 (PMC3511281; doi:10.1371/journal.pone.0050808)

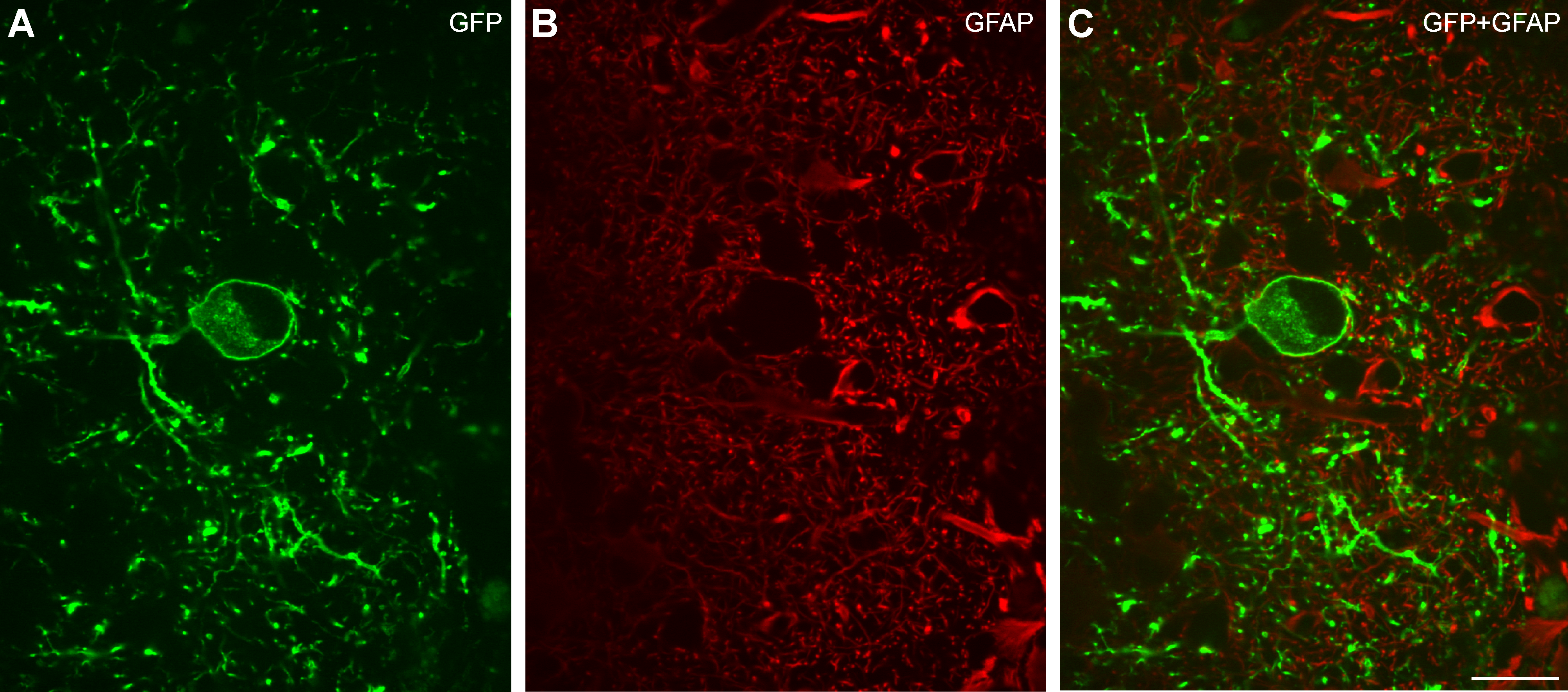

Supplement: Figure S1 — Glial elements do not express channelrhodopsins after lenti-ChR2-αEF1 transfection. Confocal fluorescence micrographs in the putamen showing eYFP/ChR2-positive (A) and GFAP-positive (glial) elements (B). ChR2-positive elements do not express GFAP, as shown in the merged imaged in (C). Scale bar: 20 µm. (TIF) [file pone.0050808.s001.tif]
